# Pleural effusion as an initial manifestation in a patient with primary pulmonary monoclonal B-cell lymphocyte proliferative disease

**DOI:** 10.1186/s12931-018-0941-6

**Published:** 2018-12-12

**Authors:** Qin Du, Lili Fan, Hongyu Zhou

**Affiliations:** 1 Department of Neurology, West China Hospital, Sichuan University, Guo Xuexiang #37, Chengdu, 610041 China; 2 Department of Respiratory, West China Hospital, Sichuan University, Guo Xuexiang #37, Chengdu, 610041 China

## To the editor,

**Fig. 1:**
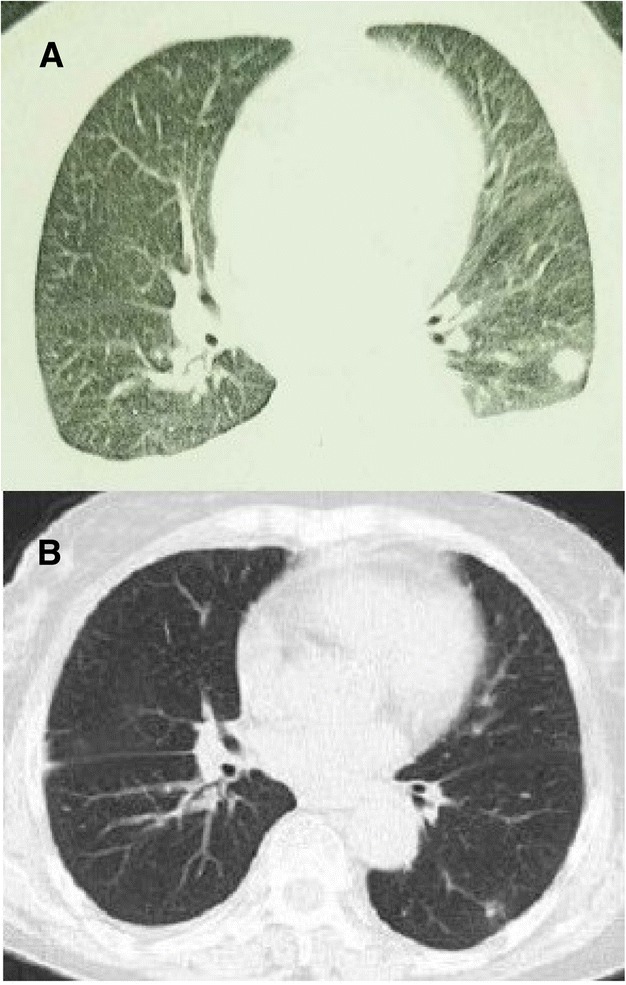
Chest CT scan in the axial plane performed 2 months following onset of symptoms showing (**a**) a nodule in the left lower lobe; the patient was administered fluconazole regularly for 4 months, and a repeated CT scan (**b**) indicated the absence of pulmonary nodule

Pleural effusion, the most common manifestation of pleural disorders, is an abnormal accumulation of fluid in the pleural cavity. In a developing country such as China, infections, particularly tuberculosis, are the predominant cause of pleural effusion [1]. Other causes, such as inflammation and malignancy, are also common. In this report, we describe a 65-year-old woman with massive bilateral pleural effusion and a left pulmonary nodule. The results of fine needle aspiration of the pulmonary nodule suggested fungal infection, likely caused by Cryptococcus. After the regular treatment of fluconazole for 4 months, the left pulmonary nodule disappeared, but bilateral pleural effusion persisted (Fig. 1). The results of phenotypic lymphocyte screening by flow cytometry of both blood and bilateral pleural effusion supported the diagnosis of primary pulmonary monoclonal B-cell lymphocyte proliferative disease. We further discuss the current understanding of this disease, including the possible pathogenesis.

In February 2017, a 65-year-old Chinese woman with no significant past medical history presented with apparent nocturnal dyspnea accompanied by a dry cough. Radiological findings revealed massive bilateral pleural effusion and a left lower pulmonary nodule. Complete positron emission tomography-computed tomography was performed (2nd May 2017) at the Second Affiliated Hospital of Xiangya, Central South University, showing that the inferior lobe of the left lung had a nodular shadow with a size of 15 × 13 mm and an obscured edge. An abnormal increase in radioactive uptake was observed. Other parts of the body showed no obviously abnormal increase in [18F]-2-fluoro-2-deoxy-D-glucose metabolism. Histological examination of the left pulmonary nodule with alkaline phosphate and periodic acid-Schiff staining indicated consolidation, proliferation of interstitial fibers, a large number of multinucleated cells, absence of necrosis, infiltration of lymphocytes and individual spores. Acid-fast staining produced negative results. These findings confirmed the diagnosis of fungal infection, with a tendency towards Cryptococcus as the cause. Immunohistochemical analysis showed CK7 (+), TTF-1 (+), CD68 (+), CD3 (+), IgG (+), CD20 (+), Ki67 (3%+), IgG4 (−), P40 (−), and CD34 (−). The patient was administered fluconazole regularly for 4 months, and a repeated CT scan indicated the absence of pulmonary nodule; however, the pleural effusion persisted. Therefore, the patient was empirically treated with antituberculosis therapy, but it failed to provide an effect. Pleural effusion was proven to be exudate, and the interferon gamma release assay, *Cryptococcus neoformans* antigen test, galactomannan test, fungus G test and tumor marker assays were negative. No carcinoma cells were detected by exfoliative cytological examination of pleural effusion. Pleural biopsy showed no evidence of either neoplasms, tuberculosis or fungal infections. To elucidate the definitive cause of pleural effusion, the patient was transferred to West China Hospital, Sichuan University, and laboratory investigation indicated no evidence of tuberculosis or immunodeficiency. The patient underwent bilateral thoracentesis; intriguingly, the chyle test of bilateral pleural effusion was positive, but the triglyceride-to-cholesterol ratio was < 1, which was a result of recurrent pleural effusion for a long time and not true chylothorax. The pleural effusion was a yellow, limpid liquid with the following counts: total protein, 49.8 g/L; karyocytes, 700 × 10^6/L; erythrocytes, 400 × 10^6/L; mononuclear cells, 81%; multinucleated cells, 19%. Contrast-enhanced high-resolution computed tomography showed strip and plaque shadows scattering bilaterally, indicating possible inflammatory lesions. Fiberoptic bronchoscopy revealed a normal lumen. Bronchoalveolar lavage fluid did not contain malignant cells. The bone marrow biopsy was normal. The painless gastroscopy and enteroscopy were normal.

**Fig. 2:**
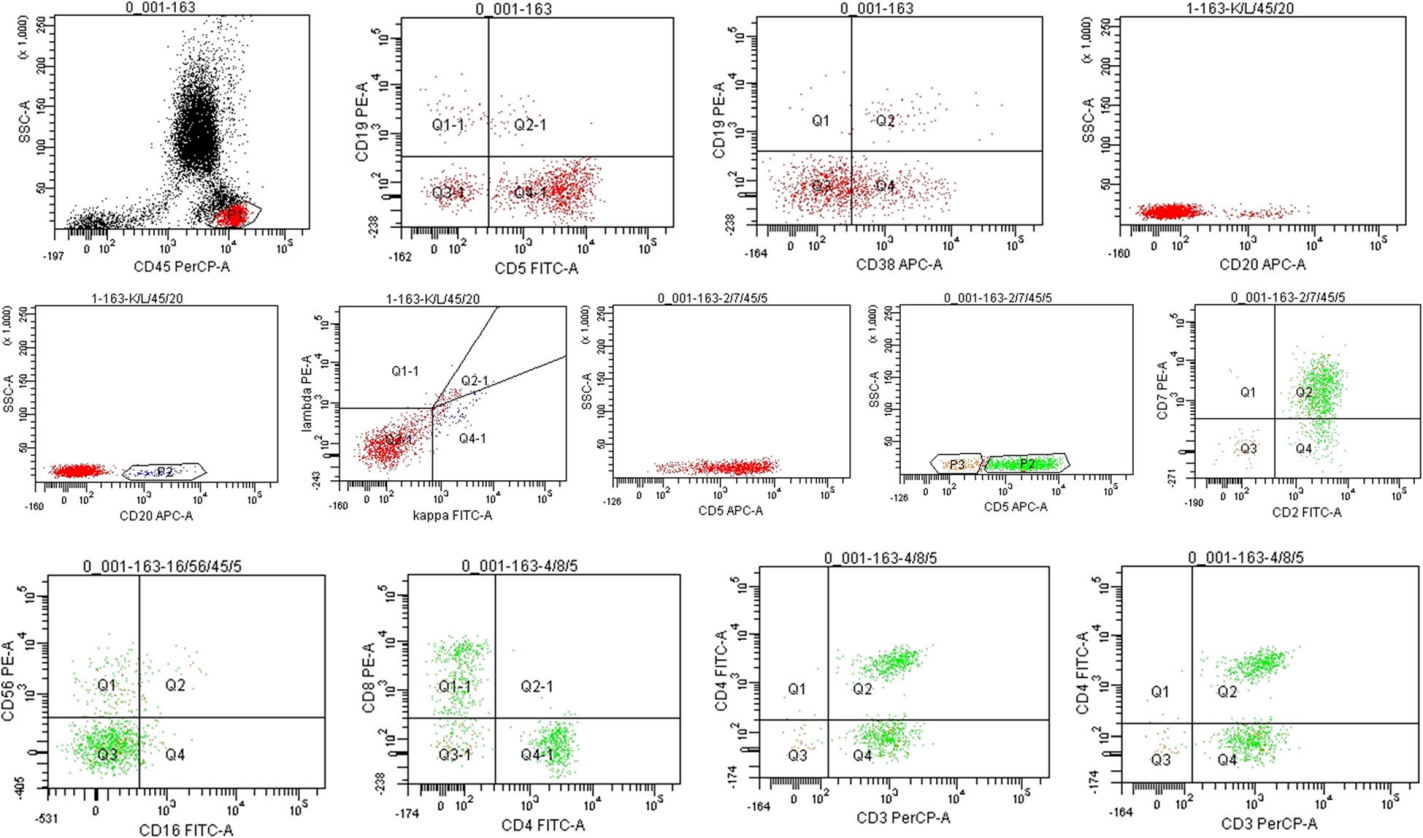
Phenotypic lymphocyte screening by flow cytometry of blood showed monoclonal B-cell lymphocyte proliferation. Lymphocytes (p1, red): 10% of nucleated cells; B cells: 6% of lymphocytes, expressing CD19, CD20 and CD38, partially expressing CD5, and restrictedly expressing kappa light chain

**Fig. 3:**
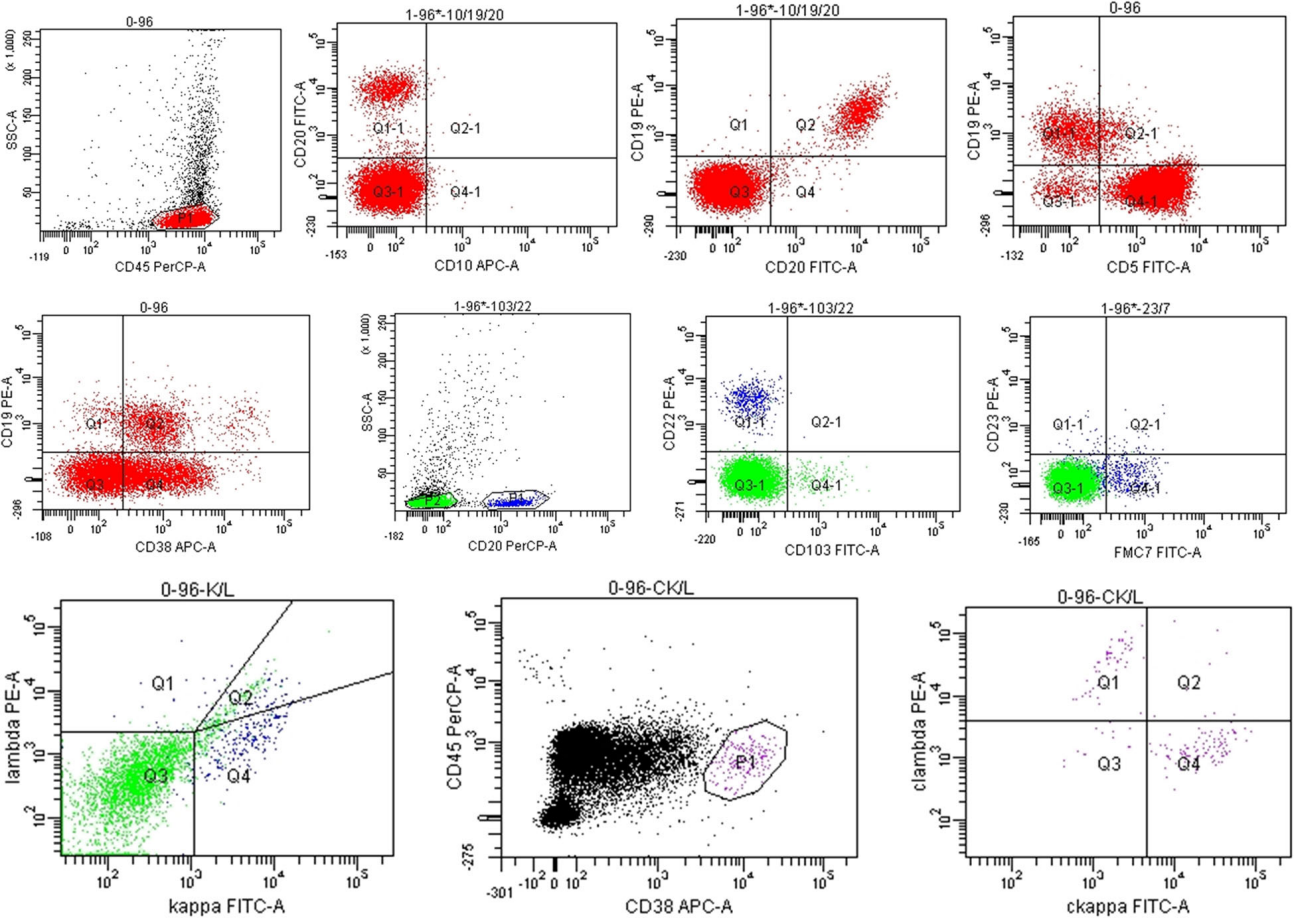
Phenotypic lymphocyte screening by flow cytometry of right pleural effusion showed monoclonal B-cell lymphocyte proliferation. Lymphocytes (p1, red): 60% of nucleated cells. B cells: 19% of lymphocytes, expressing CD19, CD20 and CD38; partially expressing CD5; and not expressing CD10. CD20(+) cells: expressing CD22, FMC7; restrictedly expressing kappa light chain; and not expressing CD23, CD103, and lambda light chain

**Fig. 4:**
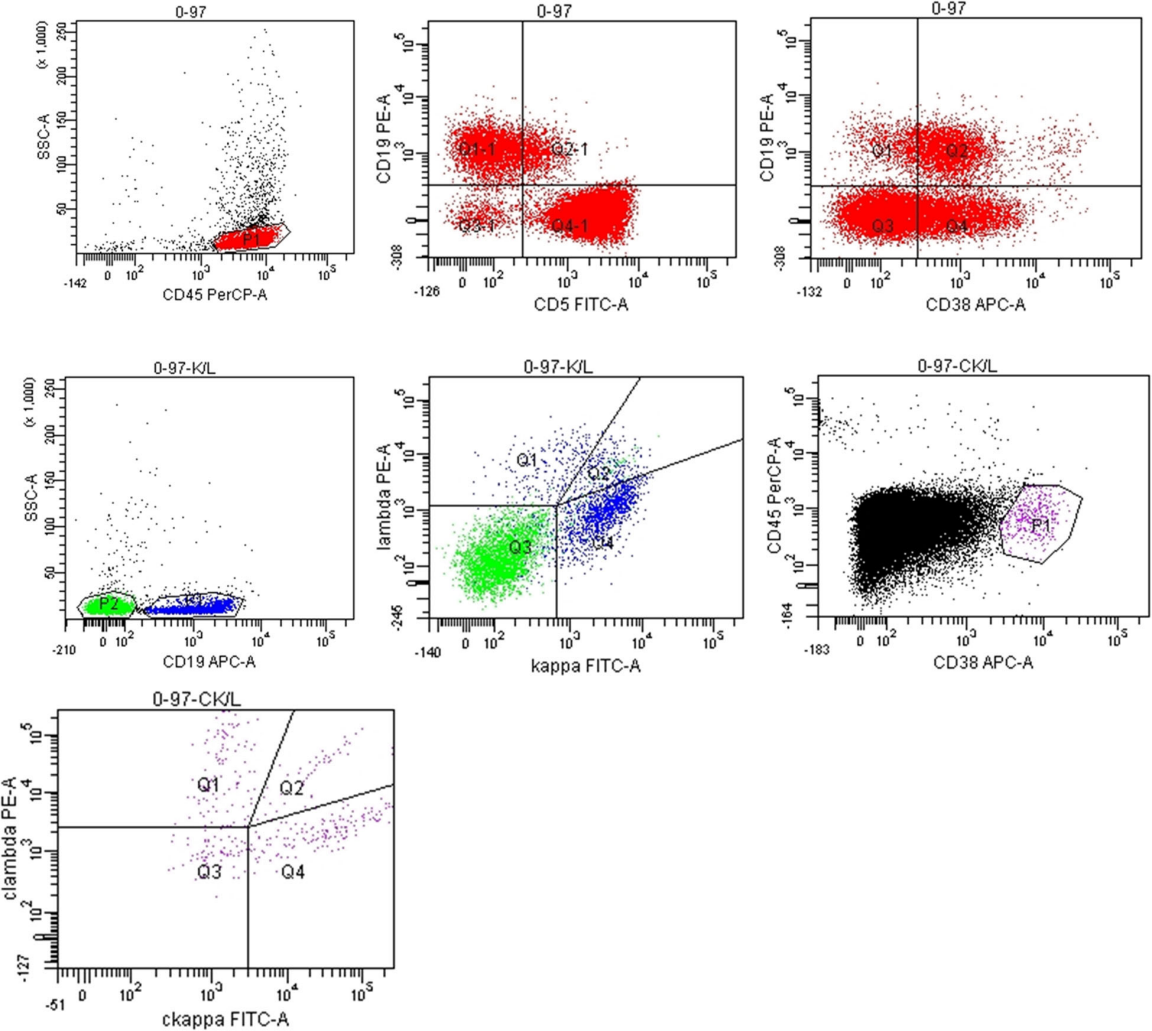
Phenotypic lymphocyte screening by flow cytometry of left pleural effusion showed monoclonal B-cell lymphocyte proliferation. Lymphocytes (p1, red): 95% of nucleated cells. B cells: 28% of lymphocytes, expressing CD19 and CD38, partially expressing CD5, and highly expressing kappa light chain, κ:λ ~ 5.3

To further proceed with the diagnosis, phenotypic lymphocyte screening by flow cytometry of both blood and bilateral pleural effusion was performed, and all of the results showed monoclonal B-cell lymphocyte proliferation (Figs. 2, 3 and 4). Based on the above findings showing no extrapulmonary involvement, primary pulmonary monoclonal B-cell lymphocyte proliferative disease was ultimately diagnosed. Due to a lack of medical support, the patient refused further treatment.

Primary pulmonary monoclonal B-cell lymphocyte proliferative disease with atypical clinical manifestation is usually a malignant hyperplastic disease of the hematological system. Herein, we describe a patient who presented with pleural effusion, according to pathological findings and treatment results, and the pulmonary nodule was confirmed to have Cryptococcus infection. Cryptococcus is more common in patients with immunodeficiency; thus, we sought to determine whether a fundamental disease such as immune deficiency was present. Because of refusal to undergo thoracoscopy, the patient underwent bilateral pleural biopsy and multiple thoracentesis. Ultimately, we established a diagnosis of primary pulmonary monoclonal B-cell lymphocyte proliferative disease based on pathology. Although this case lacked direct histopathological evidence, comprehensive examinations excluded tuberculosis and the recurrence of fungal infection. Furthermore, phenotypic lymphocyte screening by flow cytometry of blood and bilateral pleural effusion indicated monoclonal B-cell lymphocyte proliferation. Extrapulmonary diseases were also excluded; therefore, we concluded that primary pulmonary lymphoma (PPL) was likely. PPL is an extremely rare form of lymphoma with an incidence of 0.4% [2]; for the sake of making a diagnosis of PPL, extrapulmonary diseases must be ruled out [3]. Approximately half of PPL cases have no symptoms or lack specific respiratory manifestations, such as cough, dyspnea, chest pain and so on [4]. Due to its nonspecific presentation, the diagnosis of PPL is exceptionally challenging and usually leads to misdiagnosis or delayed diagnosis [5]. Radiographic appearances include patchy opacity, mediastinal masses, solitary or multiple nodules, and pleural effusion, either unilateral or bilateral. Serum protein electrophoresis anomalies are observed in 33% of patients with PPL [3]. To the best of our knowledge, clinical manifestations of PPL have various forms, but pleural effusion as the initial manifestation is extremely rare. Considering the patient’s manifestations, including recurrent pleural effusion, as well as the respiratory findings and other examinations of the various systems, malignancy remained highly suspicious in the differential diagnosis. According to a series of examinations, ultimately the diagnosis of PPL was highly suspected, but it was not confirmed by the histopathological evidence, perhaps because the patient’s plasma cells did not secrete and express some special proteins, or the patient’s condition was in the early stage of PPL and unable to show the typical clinical manifestations. Therefore, we failed to determine where the monoclonal B lymphocytes originated from. During the 10 months of follow-up, the patient’s bilateral pleural effusion volume grew slowly, which demonstrated that her underlying disease has not been eradicated. We speculate that the cause is a relatively low-grade lymphoma. The patient’s condition is currently under observation and requires long-term follow-up.

## Conclusion

Primary pulmonary monoclonal B-cell lymphocyte proliferative disease is an uncommon disease with varied atypical manifestations where pleural effusion is even rare, especially as the initial clinical manifestation. For our patient with agnogenic Cryptococcus infection, investigating the unexplained pleural effusion for other underlying causes resulted in a delayed diagnosis. There are many reasons for pleural effusion, and despite the common causes of pleural effusion such as tuberculosis, lung carcinoma and other solid tumors, nonsolid tumors such as primary pulmonary B lymphocyte monoclonal proliferative disease should also be considered.

## Abbreviations

PPL: Primary pulmonary lymphoma
